# Respiratory amyloidosis: a case series from a Brazilian referral center

**DOI:** 10.36416/1806-3756/e20240047

**Published:** 2024-05-29

**Authors:** Philippe de Figueiredo Braga Colares, Alfredo Nicodemos Cruz Santana, Rodrigo Abensur Athanazio, Ronaldo Adib Kairalla, Bruno Guedes Baldi

**Affiliations:** 1. Divisão de Pneumologia, Instituto do Coração, Hospital das Clínicas, Faculdade de Medicina, Universidade de São Paulo, São Paulo (SP) Brasil.; 2. Hospital de Base de São José do Rio Preto, Faculdade de Medicina de São José do Rio Preto, São José do Rio Preto (SP) Brasil.; 3. Hospital Regional da Asa Norte - HRAN - Curso de Medicina e Enfermagem, Escola Superior de Ciências da Saúde, Brasília (DF) Brasil.

## TO THE EDITOR:

Amyloidosis is characterized by abnormal extracellular protein aggregation and deposition, which may occur associated with inflammatory, hereditary, or neoplastic conditions and may involve a single organ or be multisystemic.^1^ ^-^ ^3^ Consequently, these patients may present with various clinical manifestations involving different organs. The International Classification of Amyloidosis^4^ recognizes 30 types of human amyloids and their precursors. Amyloidosis can be classified according to the type of amyloid, extent (local or systemic), nature of the disease (acquired or inherited), and organs involved.

Amyloidosis occurs in approximately 60% of patients with immunoglobulin light-chain amyloidosis (AL), 10% of those with serum amyloid A amyloidosis (AA) due to chronic inflammatory diseases, 10% of those with transthyretin amyloidosis (ATTR) from variations (mutations) in the transthyretin gene (mut-ATTR), 8% of those with wild-type ATTR (wt-ATTR) amyloidosis, and 10% of those with localized amyloidosis.^3^ ^-^ ^5^ Histologically, amyloid deposits appear as apple-green birefringence under polarized light with Congo red dye staining.^1^ ^-^ ^3^

Although the actual incidence of amyloidosis is unclear, it is estimated to be 10-14 cases per million person-years.^6^ Based on histopathological studies, the prevalence of pulmonary deposition in AL patients ranges from 36% to 90%.^3^

Respiratory amyloidosis may be localized or be part of systemic amyloidosis, and it may be categorized as tracheobronchial, nodular, or interstitial.^1^ ^-^ ^3^ ^,^ ^5^ Furthermore, there may be mediastinal or hilar lymphadenopathy and pleural effusion. Cystic lung disease associated with pulmonary amyloidosis is another uncommon presentation that has been described to be associated with collagen vascular diseases, mainly Sjögren’s syndrome and lymphoproliferative disease.^7^ Factors potentially related to the pattern of pulmonary deposition in amyloidosis remain unclear.

This retrospective study aimed to describe all patients with respiratory amyloidosis who were followed at the Pulmonary Division of the University of Sao Paulo, located in the city of São Paulo, Brazil, between 2010 and 2022.^1^ ^-^ ^3^ Data were collected by reviewing the medical records of patients. We evaluated demographic and clinical characteristics, comorbidities, laboratory examinations, pulmonary function tests, imaging records, histopathological characteristics, treatments, and outcomes, including survival and complications. The study protocol was approved by the local research ethics committee (CAAE no. 76830724.9.0000.0068).

Pulmonary function tests (PFTs) were performed with a calibrated pneumotachograph (Koko PFT; nSpire Health Inc., Longmont, CO, USA). The following variables were collected: FEV_1_, FVC, FEV_1_/FVC ratio, RV, TLC, RV/TLC ratio, and DL_CO_. Predicted values were derived from a Brazilian population.^8^ We also evaluated pulse oximetry at room air.

HRCT was performed in all patients, and results were classified into three patterns: nodular pulmonary, diffuse alveolar-septal, or tracheobronchial amyloidosis. All tissue biopsies were reviewed by pulmonary pathologists at the University of São Paulo. Congo red dye stained samples under polarized light showing apple-green birefringence is the gold standard for the diagnosis of amyloidosis.^1^ ^-^ ^3^ Tissue samples were obtained through bronchoscopy (n = 10), surgical biopsy (n = 5), or transthoracic biopsy (n = 5), depending on the lung pattern, after multidisciplinary discussion.

Continuous variables with normal distribution were expressed as means and standard deviations. Categorical variables were described as absolute and relative frequencies. One-way ANOVA was used to compare functional data at diagnosis with the latest available functional data, and the Tukey’s test was used when multiple comparisons were necessary. Statistical significance was set at p < 0.05. Survival was estimated using the Kaplan-Meier method. Data were analyzed using the IBM SPSS Statistics software package, version 21.0 (IBM Corporation, Armonk, NY, USA).

Twenty patients with respiratory amyloidosis were assessed and diagnosed as having tracheobronchial amyloidosis, in 9; nodular amyloidosis, in 8; and amyloidosis with interstitial patterns, in 3. Patient characteristics are presented in Table 1. The mean age at diagnosis was 56 years, and patients with interstitial patterns were younger; 55% of the patients were women, and dyspnea was the most common symptom (80%). PFTs were available for 12/20 of the patients (60%): 6 patients (30%) presented with an obstructive pattern, 2 (10%) showed a restrictive pattern, and 4 (20%) had normal PFTs.

**Table 1:** Demographics, clinical characteristics, functional features, and survival of patients with respiratory amyloidosis.^a^

Variable	Group				p
	Tracheobronchial	Nodular	Interstitial	Total	
	(n = 9)	(n = 8)	(n =3)	(N = 20)	
Diagnosis delay, years	3.7 ± 3.9	3.3 ± 2.9	4.2 ± 3.3	3.6 ± 3.3	0.933
Clinical characteristics					
Age at onset of symptoms, years	50.2 ± 12.5	59.8 ± 9.9	39.7 ± 16.0	52.5 ± 13.4	0.060
Age at diagnosis, years	54.0 ± 9.91	63.3 ± 8.70	44.0 ± 18.2	56.2 ± 12.3	0.043
Sex, female	3 (33.3)	5 (62.5)	3 (100)	11 (55.0)	0.064
Race or ethnicity, White	8 (88.9)	6 (75.0)	3 (100)	17 (85.0)	0.442
Smoker (current or former smoker)	4 (44.4)	4 (50.0)	0 (0.00)	8 (40.0)	0.177
Peripheral oximetry, %	96.0 ± 1.2	95.0 ± 2.2	97.0 ± 2.3	96.0 ± 1.9	0.226
Presence of symptoms	8 (88.9)	5 (62.5)	3 (100)	16 (80.0)	0.207
Weight loss	0 (0.0)	1 (12.5)	0 (0.0)	1 (5.0)	0.384
Dyspnea	8 (88.9)	5 (62.5)	3 (100)	16 (80.0)	0.207
Cough	3 (33.3)	3 (37.5)	3 (100)	9 (45.0)	0.064
Hemoptysis	1 (11.1)	1 (12.5)	0 (0.0)	2 (10.0)	0.706
Comorbidities	6 (66.7)	8 (100)	3 (100)	17 (85.0)	0.066
Systemic hypertension	3 (33.3)	6 (75.0)	0 (0.0)	9 (45.0)	0.029
Diabetes mellitus/dyslipidemia	2 (22.2)	3 (37.5)	0 (0.0)	5 (25.0)	0.305
Hypothyroidism	0 (0.0)	2 (25.0)	1 (33.3)	3 (15.0)	0.129
Heart failure/ischemic heart disease	3 (33.3)	2 (25.0)	0 (0.0)	5 (25.5)	0.361
Kidney disease	0 (0.0)	0 (0.0)	1 (33.3)	1 (5.0)	0.127
Depression/anxiety	1 (11.1)	0 (0.0)	1 (33.3)	2 (10.0)	0.234
Hematological disease	0 (0.0)	1 (12.5)	2 (66.7)	3 (15.0)	0.029
Sjögren’s syndrome/lymphocytic bronchiolitis	0 (0.0)	2 (25.0)	1 (33.3)	3 (15.0)	0.129
COPD	1 (11.1)	1 (12.5)	0 (0.0)	2 (10.0)	0.706
Protein electrophoresis, abnormal	2 (22.2)	3 (37.5)	2 (66.7)	7 (35.0)	0.375
Autoimmune features	1 (11.1)	2 (25.0)	1 (33.3)	4 (20.0)	0.631
Exposure to	1 (11.1)	2 (25.0)	0 (0.0)	3 (15.0)	0.442
Birds or feather/down-containing items	1 (11.1)	2 (25.0)	0 (0.0)	3 (15.0)	0.442
Mold or mildew	0 (0.0)	0 (0.0)	0 (0.0)	0 (0.0)	-
Pulmonary function tests	4 (44.4)	6 (75.0)	2 (66.7)	12 (60.0)	0.419
Obstructive pattern	3 (33.3)	2 (25.0)	0 (0.0)	5 (25.0)	0.361
FEV_1_, % of predicted	80.3 ± 11.1	51.0 ± 29.7	-	68.6 ± 23.2	0.382
Restrictive pattern	0 (0.0)	1 (12.5)	1 (33.3)	2 (10.0)	0.206
FVC, % of predicted	-	54	77	64 [52-77]	-
Survival, years	6.6 ± 4.7	7.8 ± 5.7	1.7 ± 1.2	6.3 ± 5.1	0.209

a Values expressed as n (%), mean ± SD, or median [IQR].

By definition, systemic amyloidosis is characterized by deposits of AL, AA, ATTR, and mut-ATTR proteins.^1^ ^-^ ^5^ Considering only the involvement of multiple organs, without immunohistochemistry confirmation, 1 patient had cutaneous involvement and another 1 had renal involvement. Hematological disease and Sjögren’s syndrome were present in 15% of the patients. Four patients with tracheobronchial amyloidosis underwent an endoscopic procedure, with endobronchial dilation or placement of a prosthesis (tracheostomy or T-tube). One patient with interstitial amyloidosis underwent kidney transplantation. The median survival time was 6 ± 5 years (range: 0.5-19; p = 0.209). One patient with interstitial amyloidosis died of pulmonary sepsis, and another 8 patients were lost to outpatient follow-up; therefore, their outcomes are unknown.

There were no statistically significant differences among the three groups regarding functional features and survival, and patients with nodular and tracheobronchial patterns tended to have a long evolution. Among those with autoimmune features, Sjögren’s syndrome and lymphocytic bronchiolitis were the most common. Patients with tracheobronchial or nodular amyloidosis had normal or obstructive PFT pattern more often. Furthermore, although cardiac involvement is the most common immediate cause of death in patients with pulmonary amyloidosis, only 25% of the patients reported previous cardiac impairment.

Here, we present a case series of patients with different manifestations of respiratory amyloidosis and clinical and functional impairments regardless of the type of disease. Additionally, our study did not demonstrate mediastinal lymph node or pleural amyloidosis, which differs from other studies.^8^ ^-^ ^10^ However, the survival rates of our patients were similar to those in previous studies.^1^ ^,^ ^5^ There is no definitive treatment for respiratory amyloidosis, and supportive care is the main target in the management of the disease to decrease symptoms and improve quality of life.

The major limitations of our study were its retrospective nature and the small number of patients included. However, amyloidosis is a rare disease; therefore, we believe that we have a relevant number of patients in our study. Another limitation was that the analysis of amyloidotic material with the proteomic method of mass spectrometry (the new gold standard for fibril typing) was not performed.

In conclusion, respiratory amyloidosis may have different manifestations and should be considered in the differential diagnosis of patients with tracheobronchial involvement, pulmonary nodules, or interstitial lung abnormalities. Furthermore, most patients with respiratory amyloidosis are asymptomatic and require careful follow-up.
